# Molecular detection of *Coxiella burnetii* infection in small mammals from Moshi Rural and Urban Districts, northern Tanzania

**DOI:** 10.1002/vms3.401

**Published:** 2020-12-05

**Authors:** Ndyetabura O. Theonest, Ryan W. Carter, Elizabeth Kasagama, Julius D. Keyyu, Gabriel M. Shirima, Rigobert Tarimo, Kate M. Thomas, Nick Wheelhouse, Venance P. Maro, Daniel T. Haydon, Joram J. Buza, Kathryn J. Allan, Jo E.B. Halliday

**Affiliations:** ^1^ School of Life Sciences and Bioengineering Nelson Mandela African Institution of Science and Technology Arusha Tanzania; ^2^ Kilimanjaro Clinical Research Institute Moshi Tanzania; ^3^ The Boyd Orr Centre for Population and Ecosystem Health Institute of Biodiversity Animal Health and Comparative Medicine College of Medical Veterinary and Life Sciences University of Glasgow Glasgow UK; ^4^ Tanzania Wildlife Research Institute Arusha Tanzania; ^5^ Centre for International Health Dunedin School of Medicine University of Otago Dunedin New Zealand; ^6^ School of Applied Sciences Edinburgh Napier University Edinburgh UK; ^7^ Kilimanjaro Christian Medical University College Moshi Tanzania

**Keywords:** *Coxiella burnetii*, detection, prevalence, small mammal, Tanzania, zoonoses

## Abstract

*Coxiella burnetii* is an obligate intracellular bacterium that causes Q fever, a zoonotic disease of public health importance. In northern Tanzania, Q fever is a known cause of human febrile illness, but little is known about its distribution in animal hosts. We used a quantitative real‐time PCR (qPCR) targeting the insertion element IS1111 to determine the presence and prevalence of *C*. *burnetii* infections in small mammals trapped in 12 villages around Moshi Rural and Moshi Urban Districts, northern Tanzania. A total of 382 trapped small mammals of seven species were included in the study; *Rattus rattus* (*n* = 317), *Mus musculus* (*n* = 44), *Mastomys natalensis* (*n* = 8), *Acomys wilson* (*n* = 6), *Mus minutoides* (*n* = 3), *Paraxerus flavovottis* (*n* = 3) and *Atelerix albiventris* (*n* = 1). Overall, 12 (3.1%) of 382 (95% CI: 1.6–5.4) small mammal spleens were positive for *C*. *burnetii* DNA. *Coxiella burnetii* DNA was detected in five of seven of the small mammal species trapped; *R*. *rattus* (*n* = 7), *M*. *musculus* (*n* = 1), *A*. *wilson* (*n* = 2), *P*. *flavovottis* (*n* = 1) and *A. albiventris* (*n* = 1). Eleven (91.7%) of twelve (95% CI: 61.5–99.8) *C*. *burnetii* DNA positive small mammals were trapped within Moshi Urban District. These findings demonstrate that small mammals in Moshi, northern Tanzania are hosts of *C*. *burnetii* and may act as a source of *C*. *burnetii* infection to humans and other animals. This detection of *C*. *burnetii* infections in small mammals should motivate further studies into the contribution of small mammals to the transmission of *C*. *burnetii* to humans and animals in this region.

## Graphical abstract

We detected *Coxiella burnetii* in five of seven small mammals species sampled in northern Tanzania. The overall prevalence was 3.1% (12 of 382) and DNA was detected in *R. rattus*, *M. musculus*, *A. wilson*, *P. flavovottis* and *A. albiventris*. The detection of *Coxiella burnetii* in small mammals trapped in an area where this bacterium is a known cause of human febrile illness provides a clear rationale for further investigation of the epidemiology of *C. burnetti* in this setting and the role that small mammals play in multi‐host epidemiology.

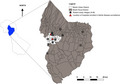

## 1 INTRODUCTION

*Coxiella burnetii* is an obligate intracellular bacterium, the causative agent of Q fever, a zoonotic disease of public health importance worldwide except in New Zealand (Marrie et al., 2015; Schimmer et al., 2014; Toman et al., 2009). *C. burnetii* can infect a wide range of vertebrate and invertebrate hosts. Domestic ruminants (sheep, goats and cattle) are considered the main reservoirs of *C*. *burnetii* (Duron et al., 2015; Van den Brom & Vellema, 2009).

In recent years, an increasing number of studies have reported the detection of *C*. *burnetii* in small mammals. Investigation of patients in the Netherlands indicated an association between small mammal sightings and Q fever case occurred during the 2007 outbreak (Karagiannis et al., 2009). Similarly, wild rodents, marsupials, bats and other wild mammals captured around the houses of Q fever case patients in French Guiana were more likely to test *C*. *burnetii* positive as compared to animals trapped at greater distance from residential houses, and sighting of these animals especially rodents was identified as a risk factor for human *C*. *burnetii* infection (Gardon et al., 2001).

Data on the presence and prevalence of *C. burnetii* in small mammals and their epidemiology in Tanzania are limited. Globally, studies on the presence and prevalence of *C*. *burnetii* in small mammals and other animals have demonstrated significant variation in the prevalence of *C. burnetii* depending on factors such as species, sex, age, season of sampling (wet or dry) and sampling location (Foronda et al., 2015; Gardon et al., 2001; Webster et al., 1995; Yadav et al., 2019). In many African countries, there are few studies on *C*. *burnetii* presence and data on prevalence in both animal and humans are scarce (Salifu et al., 2019). Based on conventional PCR detection methods targeting *C*. *burnetii* 16rRNA and IS1111 genes, the overall prevalence of *C*. *burnetii* in African small mammal populations has been found to range from 2.1% (4/194) in peridomestic rodents in Nigeria (Kamani et al., 2018) to 45% (9/20) in Zambia (Chitanga et al., 2018).

Zoonotic infections are of great importance to public health in many parts of the world but their clinical importance is typically under‐appreciated (Angelakis et al., 2014; Crump et al., 2013). *C. burnetii* infection was diagnosed in 5.0% of 482 febrile patients tested in a retrospective study performed in Moshi, northern Tanzania (the same area as this study) (Crump et al., 2013). This finding together with several recent outbreaks of Q fever highlights the importance of *C. burnetii* as a public health problem and need for continued efforts to identify reservoirs of *C. burnetii* to achieve better control and prevention.

Several PCR‐based diagnostic methods have been successfully applied for the direct detection of *C*. *burnetii* (Herrin et al., 2011; Kersh et al., 2010; Klee et al., 2006; Piñero et al., 2014; Schneeberger et al., 2010). The use of quantitative real‐time PCR (qPCR), targeting the IS1111 insertion element which is present in multiple copies, has been reported to be highly sensitive for the detection of *C*. *burnetii* DNA (Bruin et al., 2011). In this study, we aimed to use a qPCR assay targeting the transposase gene of insertion element IS1111 to determine the presence and prevalence of *C*. *burnetii* DNA in spleen tissue samples of small mammals from Moshi Rural and Urban Districts, northern Tanzania.

## 2 MATERIALS AND METHODS

### 2.1 Study site

**FIGURE 1:**
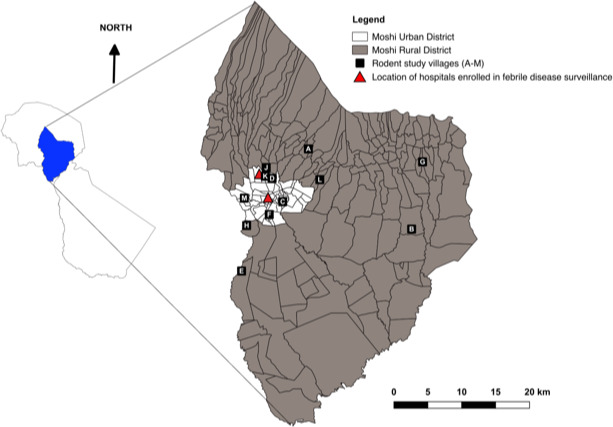
Map of Moshi Urban and Moshi Rural Districts, showing representative locations of small mammal study villages. Letters indicate the different villages in which small mammal trapping was conducted. Polygons in the main image show local administrative boundaries. Insert map on left shows outline of the Kilimanjaro region of Tanzania and the location of study districts within the region. This figure is adapted from a version published previously (Allan et al., 2018)

The study was conducted in the Kilimanjaro Region of northern Tanzania. Trapping of the small mammals was conducted in two of seven districts of Kilimanjaro Region in a previous study (Allan et al., 2018). The two districts, Moshi Municipal (Urban) and Moshi Rural (Figure 1), were chosen as the study site due to the previous finding of a high prevalence of Q fever in febrile patients from this area (Crump et al., 2013). The climate in the study area is tropical with an average temperature for the year of 74.2°F (23. 4°C) and two patterns of rains; long rains from March to May and short rains from October to December. The coolest months coinciding with the long dry season from June to September. The warmest month, on average, is February with an average temperature of 77.9°F (25.5°C). The coolest month on average is July, with an average temperature of 69.3°F (20.7°C) (Climate‐data.org, 2020). Subsistence farming is common. Agriculture, which is mainly mixed cropping and livestock farming, is the main economic activity in the study area.

### 2.2 Small mammal sampling and data collection

Small mammal spleen samples for this study were obtained from a previous cross‐sectional study (Allan et al., 2018) for which small mammal sampling was conducted within Moshi Rural and Urban Districts. Small mammals were trapped from a total of seven villages within Moshi Rural District and five villages within Moshi Urban District (Table 1 and Figure 1). The villages were randomly selected from a list of villages that were home to people that had sought care, and had been enrolled in previous febrile illness surveillance studies at local hospitals (Crump et al., 2013). As in the previous study, the target sample size was 50 small mammals per sub‐village to give power (*α* = 0.95, *β* = 0.8) to estimate *Leptospira* (Allan et al., 2018) and *C*. *burnetii* infection prevalence of 10%.

**TABLE 1:** Categorical variable summaries and *C. burnetii* qPCR IS1111 for small mammals trapped from Moshi, Tanzania (*n* = 382)

Variable		Number of small mammals tested for *C*. *burnetii*	*C*. *burnetii* positive *n* (%)	95% CI
Village Code	A	12	0 (0.0)	0.0–26.5
	B	13	1 (7.7)	0.2–36
	C	31	0 (0.0)	0.0–11.2
	D	26	7 (26.9)	11.6–47.8
	E	39	0 (0.0)	0.0–9.0
	F	109	2 (1.8)	0.2–6.5
	G	15	0 (0.0)	0.0–21.8
	H	35	0 (0.0)	0.0–10.0
	J	19	0 (0.0)	0.0–17.6
	K	23	0 (0.0)	0.0–14.8
	L	22	0 (0.0)	0.0–15.4
	M	38	2 (5.3)	0.6–17.7
District	Rural	155	1 (0.6)	0.02–3.5
	Urban	227	11 (4.8)	2.4–8.5
Sex	Male	163	7 (4.3)	1.7–8.6
	Female	219	5 (2.3)	0.7–5.2
Age	Mature	225	10 (4.4)	2.2–8.0
	Immature	157	2 (1.3)	0.2–4.5
Season	Wet	266	9 (3.4)	1.6–6.3
	Dry	116	3 (2.6)	0.5–7.4
Overall	NA	382	12 (3.1)	1.6–5.4

Small mammal trapping, identification and sampling are as previously described in another study (Allan et al., 2018). Data gathered for every trapped small mammal included: species (determined by observation of phenotypic characteristics and measurement of morphometric features), sex and reproductive maturity status (mature or immature determined based on external sexual characteristics) (Allan et al., 2018). Spleen tissues previously stored at −80°C were retrieved for the extraction of DNA used in this study.

### 2.3 DNA extraction

DNA was extracted from approximately 10 milligrams (mg) of spleen tissue (previously heat treated in PBS at 67°C for 1 hr) using the DNeasy Blood and Tissue Kit spin‐column protocol for DNA purification from tissues (Qiagen) performed in a biological safety cabinet (NuAire) at Kilimanjaro Clinical Research Institute Biotechnology Laboratory in Moshi, Tanzania. DNA was eluted in 100 µl of AE buffer and quantified using a Nano‐Drop spectrophotometer (Thermo Scientific). A no‐template extraction control (PCR‐grade water) was included for every 20 samples. DNA extracts were stored at −20°C prior to testing. To minimize the potential for qPCR inhibition due to the high concentration of host DNA, extracts were diluted in 20 µl of AE buffer to a standard DNA concentration of 10–50 ng/µl for qPCR testing.

### 2.4 Determination of sensitivity and limit of detection of the IS1111 qPCR assay

Initial set‐up and verification of the assay was performed on the Rotor‐Gene Q/6000 System (Qiagen). The approximate sensitivity and limit of detection (LoD) of the IS1111 qPCR assay for this study (295 bp target) was determined using a 10‐fold dilution series of DNA from *C*. *burnetii* Nine Mile RSA493 reference strain. The primers and probe were as follows: Forward primer (5ˈ‐CATCACATTGCCGCGTTTAC‐3ˈ), Reverse primer (5ˈ‐GGTTGGTCCCTCGACAACAT‐3ˈ), and 6‐carboxyfluorescein FAM‐labelled probe (5ˈ‐AATCCCCAACAACACCTCCTTATTCCCAC‐BHQ1‐3ˈ) as described in previous study (Roest et al., 2011).

### 2.5 IS1111 qPCR for detection of *C*. *burnetii* DNA in small mammal spleens

DNA extracts from small mammal spleens were screened for the presence of *C*. *burnetii* by qPCR using the same primers and probe as described above. The qPCR reactions were carried out in total volumes of 20 µl comprising of 10 μl QuantiNova qPCR mix (Qiagen), 0.8 μl of each primer (10 µM) and probe (5 µM), 2.6 µl nuclease‐free water and 5 μl DNA template. Positive controls (Nine Mile RSA493 strain), extraction controls (AE buffer) and no template controls (PCR‐grade water) were included in each qPCR run. Assays were performed on a Rotor‐Gene Q/6000 with thermocycling conditions as follows: 1 cycle of 95°C for 2 min followed by 45 cycles of 95°C for 5 s then 60°C for 5 s. Fluorescence readings were acquired via the green (510 nm) detection channel at the end of each annealing/extension phase. A qPCR run was considered valid when the negative controls showed no amplification and the positive controls amplified with Ct value < 40. Samples were tested in duplicate initially and then in an additional three wells if amplification (Ct < 40) was seen in one of two initial duplicate wells. A sample was considered positive for *C*. *burnetii* if at least two test wells, out of the maximum five, produced amplification with Ct < 40 and all other assay conditions were fulfilled.

### 2.6 Statistical analysis

Statistical analyses were performed in R (R Development Core Team, 2018). Binomial proportions and 95% confidence intervals for prevalence estimates were calculated using the package “binom” version 1.0‐5 for selected variables (Dorai‐Raj, 2014).

## 3 RESULTS

### 3.1 Sample characteristics

A total of 382 spleen samples from small mammals were available for testing. The majority (*n* = 317, 83. 0%) were from indigenous black rats (*Rattus rattus*). Other small mammal species tested included: house mice (*Mus musculus*, *n* = 44, 11.5%); multimammate mice (*Mastomys natalensis*, *n* = 8, 2.1%); spiny mice (*Acomys wilson*, *n* = 6, 1.6%); African pygmy mice (*Mus minutoides*, *n* = 3, 0.8%); striped bush squirrels (*Paraxerus flavovittis*, *n* = 3, 0.8%) and the four‐toed hedgehog (*Atelerix albiventris*, *n* = 1, 0.2%). Of the tested small mammal population, 219 individuals (57.3%) were female. Based on examination of external sexual characteristics, 224 (58.6%) were classified as sexually mature. The majority of small mammals (*n* = 266, 69.6%) were trapped during the wet season. Of the tested small mammal population, 227 (59.4%) were trapped from Moshi Urban District.

### 3.2 IS1111 limit of detection and prevalence of *C*. *burnetii* in spleen samples from small mammals

The assay limit of detection, with 100% reproducibility was estimated at approximately 10 genome copies per µl. *C*. *burnetii* DNA was detected by IS1111 qPCR in a total of 12 (3.1%) of 382 (95% CI: 1.6–5.4) spleen samples from small mammals. This included positive individuals from five of seven of the species tested; *Rattus rattus* (*n* = 7), *Mus musculus* (*n* = 1), *Acomys wilson* (*n* = 2), *Paraxerus flavovottis* (*n* = 1) and *Atelerix albiventris* (*n* = 1). Eleven (91.7%) of twelve (95% CI: 61.5–99.8) *C*. *burnetii* positive small mammals were trapped within Moshi Urban District. Five (2.3%) of 219 females (95% CI: 0.7–5.4) and seven (4.3%) of 163 males (95% CI: 1.7–8.6) were positive for *C*. *burnetii*. Nine (3.4%) of 266 (95% CI: 1.6– 6.3) small mammals sampled during the wet season were *C*. *burnetii* positive and three (2.6%) of 116 (95% CI: 0.5–7.4) small mammals sampled during the dry season were *C*. *burnetii* positive (Table 1).

## 4 DISCUSSION

*C. burnetii* DNA was detected in 3.1% of small mammals trapped from Moshi Rural and Moshi Urban Districts in northern Tanzania between May 2013 and September 2014. To the best of our knowledge, this is the first study to demonstrate the presence and prevalence of *C*. *burnetii* in small mammals from Tanzania. Infected small mammals may act as a source of *C*. *burnetii* infection for both humans and other animals in the study area. The findings of this study provide evidence to inform Q fever control programs.

There is a significant variation in the prevalence of *C*. *burnetii* reported in different small mammal populations and at different locations within Africa (Abdel‐Moein & Hamza, 2018; Chitanga et al., 2018; Kamani et al., 2018). Knowledge of the prevalence of *C*. *burnetii* in different small mammal populations and an improved understanding of the factors that drive this variation will be important to inform the design of *C*. *burnetii* control programs.

In this study, there are indications of variation in the proportion of small mammals that are *C*. *burnetii* positive across different small mammal species, season of sampling, age, species, sex and location of sampling (rural vs. urban districts and villages) (Table 1 and 2). The small number of positive individuals identified in this study limits the scope for statistical analyses of these patterns, but the factors that determine prevalence in these populations should be investigated further. In this study, the majority of *C*. *burnetii* positive small mammals were trapped from Moshi Urban District. Previous studies have suggested that emerging and re‐emerging zoonotic diseases and pathogens are linked with increasing globalization and urbanization (Amitai et al., 2010; Buzan et al., 2017; Comer et al., 2001) and there is a clear rationale for further investigation of the links between urbanization and *C. burnetii* prevalence. In this study, the small mammals sampled were trapped in or around households, indicating a potential risk of *C*. *burnetii* transmission to humans, pets and livestock.

**TABLE 2:** *Coxiella burnetii* IS1111 qPCR‐positive samples and the characteristics of positive small mammal trapped from Moshi Urban and Rural districts, Tanzania (*n* = 12)

Small mammal sample ID	Sex	Species	Locations	Season	Age	Ct Values		
						Ct1	Ct2	Av.Ct
R0024	Male	*R. rattus*	Rural	Wet	Mature	32.22	31.78	32.00
R0062	Male	*A. wilson*	Urban	Wet	Mature	30.56	30.87	30.72
R0063	Female	*A. wilson*	Urban	Wet	Mature	27.16	32.35	29.76
R0065	Female	*R. rattus*	Urban	Wet	Mature	32.59	32.84	32.72
R0067	Male	*R. rattus*	Urban	Wet	Immature	33.65	34.86	34.26
R0078	Male	*A. albiventris*	Urban	Wet	Mature	36.99	34.27	35.63
R0083	Male	*R. rattus*	Urban	Wet	Mature	31.32	32.11	31.72
R0084	Female	*R. rattus*	Urban	Wet	Mature	35.17	35.09	35.13
R0168	Male	*R. rattus*	Urban	Wet	Mature	27.90	27.76	27.83
R0330	Female	*R. rattus*	Urban	Dry	Mature	20.01	20.06	20.04
R0339	Female	*R. rattus*	Urban	Dry	Mature	27.40	28.54	27.97
R0393	Male	*R. rattus*	Urban	Dry	Immature	27.40	27.54	27.47

Observations from previous studies and the raw data from this one indicate that specific small mammal species appear more likely to carry and maintain *C*. *burnetii* bacteria than others in a given geographical area (Burgdorfer et al., 1963; Reusken et al., 2011; Rozental et al., 2017). A study to assess susceptibility of rodent species to *C. burnetii* and other rickettsiae species indicated that variation in host genetic factors that determine macrophage responses, the infecting strain of *C. burnetii* and the route of infection may explain variation in *C. burnetii* infection prevalence in small mammal (Rehácek et al., 1992). Similarly, more *C*. *burnetii* positive individuals were classified as mature small mammals as compared to immature, consistent with previous findings of increased *C. burnetii* infection in mature mice (Leone et al., 2007). Q fever and a number of other bacterial infections are typically considered as diseases of mature adults due to age‐associated physiological and anatomical changes, and dysfunction of the immune system (Gavazzi & Krause, 2002). More male small mammals trapped in this study were *C*. *burnetii* positive as compared to females, also consistent with previous findings (Thompson et al., 2012). Male small mammals have been demonstrated to exhibit frequent and long‐distance movements in search of female mates or defence of their territory. This behaviour may increase their risk of acquiring *C. burnetii* infection from the environment or from their multiple mates (Adler, 2011; Kozakiewicz et al., 2007; Nelson, 1995).

Studies conducted in the Netherlands, suggest a role for rodents in maintaining the cycle of *C*. *burnetii* infection between wildlife and domestic animals, and consequently transmission to humans (Reusken et al., 2011). Similar *C*. *burnetii* transmission scenarios may be happening in Tanzania, where the main source for human *C*. *burnetii* infection is poorly understood. In the USA and Canada *C*. *burnetii* has been detected in small mammal species trapped in the forest and pristine environments, where human activities such as livestock keeping do not occur, suggesting that small mammals in these livestock‐free areas could be acting as a reservoir of *C*. *burnetii* (Burgdorfer et al., 1963; Thompson et al., 2012).

## 5 CONCLUSIONS

In Tanzania febrile illnesses caused by zoonotic pathogens, including *C*. *burnetii,* are of public health importance but are often underappreciated or misdiagnosed. In this study, we demonstrate the detection of *C*. *burnetii* in small mammals trapped in and around household premises from the same area where a previous study has reported high prevalence of Q fever in humans. These data provide a clear rationale for further investigation of the epidemiology of *C*. *burnetti* in this setting and the role that small mammals play in this multi‐host epidemiology. Additional work is needed to understand the role of small mammals in the maintenance and transmission of *C. burnetii* infection in this region of Tanzania and to examine linkages between human, livestock and small mammal infections. *C. burnetii* strains circulating in small mammals should be typed and compared with isolates from human, other animals and environmental sources. This will provide information on the role of small mammals in *C*. *burnetii* transmission.

## 6 ETHICS STATEMENT

Approval for the study was granted by the Tanzania Commission for Science and Technology (COSTECH 2012‐471‐ER‐2005‐141 & 2015‐71‐NA‐2011‐199); Kilimanjaro Christian Medical Centre (KCMC) Ethics Committee (535 & 537); National Institute of Medical Research (NIMR), Tanzania (NIMR/HQ/R.8a/Vol.IX/1499 & NIMR/HQ/R.8a/Vol.IX/1522); Tanzania Wildlife Research Institute (TAWIRI); University of Glasgow College of Medical, Veterinary and Life Sciences Ethics Committee (200,120,020), and University of Glasgow Faculty of Veterinary Medicine Ethics and Welfare Committee (01a/13 & 02a/13). Written consent for study participation was obtained for each participating household, using forms translated into Swahili (Allan et al., 2018). Small mammal sampling was performed in accordance with the UK Guidance on the Operation of the Animals (Scientific Procedures) Act 1986 and American Veterinary Medical Association Guidelines for the Euthanasia of Animals (Home Office, 2014; AVMA Panel on Euthanasia, 2013).

## CONFLICT OF INTEREST

The authors have no conflict of interests concerning the work reported in this manuscript.

## AUTHOR CONTRIBUTION

**NDYETABURA O THEONEST:** Conceptualization; Data curation; Formal analysis; Investigation; Methodology; Writing‐original draft; Writing‐review & editing. **Ryan W Carter:** Data curation; Investigation; Methodology; Writing‐review & editing. **Elizabeth Kasagama:** Investigation; Methodology; Writing‐review & editing. **Julius D Keyyu:** Conceptualization; Funding acquisition; Project administration; Supervision; Writing‐review & editing. **Gabriel Mkilema Shirima:** Conceptualization; Funding acquisition; Investigation; Supervision; Writing‐review & editing. **Rigobert Tarimo:** Investigation; Methodology; Writing‐review & editing. **Kate M Thomas:** Data curation; Investigation; Methodology; Validation; Writing‐review & editing. **Nick Wheelhouse:** Data curation; Supervision; Validation; Writing‐review & editing. **Venance P Maro:** Conceptualization; Funding acquisition; Investigation; Project administration; Supervision; Writing‐review & editing. **Daniel T Haydon:** Conceptualization; Funding acquisition; Investigation; Project administration; Supervision; Writing‐review & editing. **Joram J Buza:** Conceptualization; Funding acquisition; Investigation; Project administration; Supervision; Writing‐review & editing. **Kathryn J Allan:** Conceptualization; Data curation; Funding acquisition; Investigation; Methodology; Project administration; Resources; Supervision; Validation; Visualization; Writing‐review & editing. **Jo E. B. Halliday:** Conceptualization; Data curation; Funding acquisition; Investigation; Methodology; Project administration; Resources; Supervision; Validation; Visualization; Writing‐review & editing.

### Peer Review

The peer review history for this article is available at https://publons.com/publon/10.1002/vms3.401.

## Data Availability

The data that support the findings of this study will be openly available after publication through: http://dx.doi. org/10.5525/gla.researchdata.948.
